# New insight into the features of Behçet’s disease with gastrointestinal ulcer: a cross-sectional observational study

**DOI:** 10.1186/s13023-021-02056-0

**Published:** 2021-10-21

**Authors:** Jing-Fen Ye, Cheng-Cheng Hou, Hua-Fang Bao, Jian-Long Guan

**Affiliations:** 1 Department of Immunology and Rheumatology Division, Fudan University Huadong Hospital, #221 Yan’an West Road, Shanghai, 200040 People’s Republic of China; 2 Division of Immunology and Rheumatology, Shanghai Key Laboratory of Clinical Geriatric Medicine, Research Center on Aging and Medicine, Huadong Hospital, Fudan University, No. 221 West Yan’an Road, Shanghai, 200040 People’s Republic of China

**Keywords:** Behçet’s disease, Gastrointestinal ulcer, Clinical characteristics, Risk factors

## Abstract

**Background:**

Behçet’s disease (BD) can involve any site of the alimentary canal. There has been research concerning intestinal BD. Nevertheless, the entire digestive tract not yet been studied extensively. Therefore, the purpose of study was to describe the prevalence, location, clinical features and possible risk factors of BD with gastrointestinal tract ulcer.

**Methods:**

This was a cross-sectional observational study that included 1232 consecutive BD patients who routinely underwent endoscopy upon their wishes. The clinical symptoms, endoscopic findings, and histologic features of BD with gastrointestinal ulcer and negative *Helicobacter pylori* (Hp) were identified.

**Result:**

We found that 22.16% (273/1232) BD patients had ulcers of the alimentary tract. At presentation, 61.54% (168/273) patients were asymptomatic. Isolated gastroduodenal involvement is an extremely usual event. The second was the pairwise combination between bowel segments, and 24 cases involved three segments at the same time. One patient suffered from total gastrointestinal tract involvement. Inflammation was the most common histopathologic feature 77.60% (142/183). The 273 BD patients with gastrointestinal ulcer were at greater risk of having archenteric symptoms (OR 0.070, *P* < 0.001), fever (OR 0.115, *P* = 0.047), high CRP (OR 0.994, *P* = 0.027) and BDCAF level (OR 0.590, *P* = 0.010). Uveitis correlates negatively with gastrointestinal involvement in BD patients (OR 3.738, *P* = 0.011).

**Conclusions:**

BD could affect the upper gastrointestinal tract independently. Endoscopy should be conducted in all patients in whom a diagnosis of BD is entertained, especially in patients with higher CRP, disease activity and fever. While, BD patients with uveitis correlates negatively with gastrointestinal involvement.

## Introduction

Behçet’s disease (BD) is a systemic vasculitis that can involve various organs at temporally distinct phases. Digestive tract ulcer, a common manifestation of BD, may develop about 4.5–6 years after the onset of oral ulcerations [1]. Despite the fact that the ileocecal region is the part most often involved, involvement of the upper gastrointestinal tract and small intestine have also been reported [2–5]. To date, much research has been conducted to investigate the involvement of the lower gastrointestinal tract; however, the involvement of the entire gastrointestinal in BD has not been studied sufficiently. Clinical presentation varies, with the most common symptom of intestinal BD being abdominal pain with or without diarrhea. About 30% of intestinal BD patients present with emergency conditions such as hemorrhage or perforation [6]. The clinical manifestations of upper gastrointestinal tract involvement may present with odynophagia and substernal pain. A study showed that the prevalence of esophageal involvement in BD is very uncommon and that patients with such involvement face few complications [7]. The incidence of gastrointestinal tract involvement in BD is inconsistently reported, possibly because of population region or research criteria [6]. Another reason is that gastrointestinal ulcers frequently appear in asymptomatic patients. In a previous study, colonoscopy was performed in 401 consecutive BD subjects, 88 with intestinal lesions. Among the subjects with ulcers, 62.86% did not have any abdominal discomfort [8]. In routine practice, endoscopy is performed in BD patients merely with symptoms. Therefore, we suspect that the real frequency of gastrointestinal BD might be higher. The present study was designed to determine the prevalence, location, clinical features of digestive tract ulcers; we determined risk factors that predicted gastrointestinal involvement in consecutive BD patients undergoing endoscopy, irrespective of gastrointestinal symptoms.

## Methods and patients

**Fig. 1:**
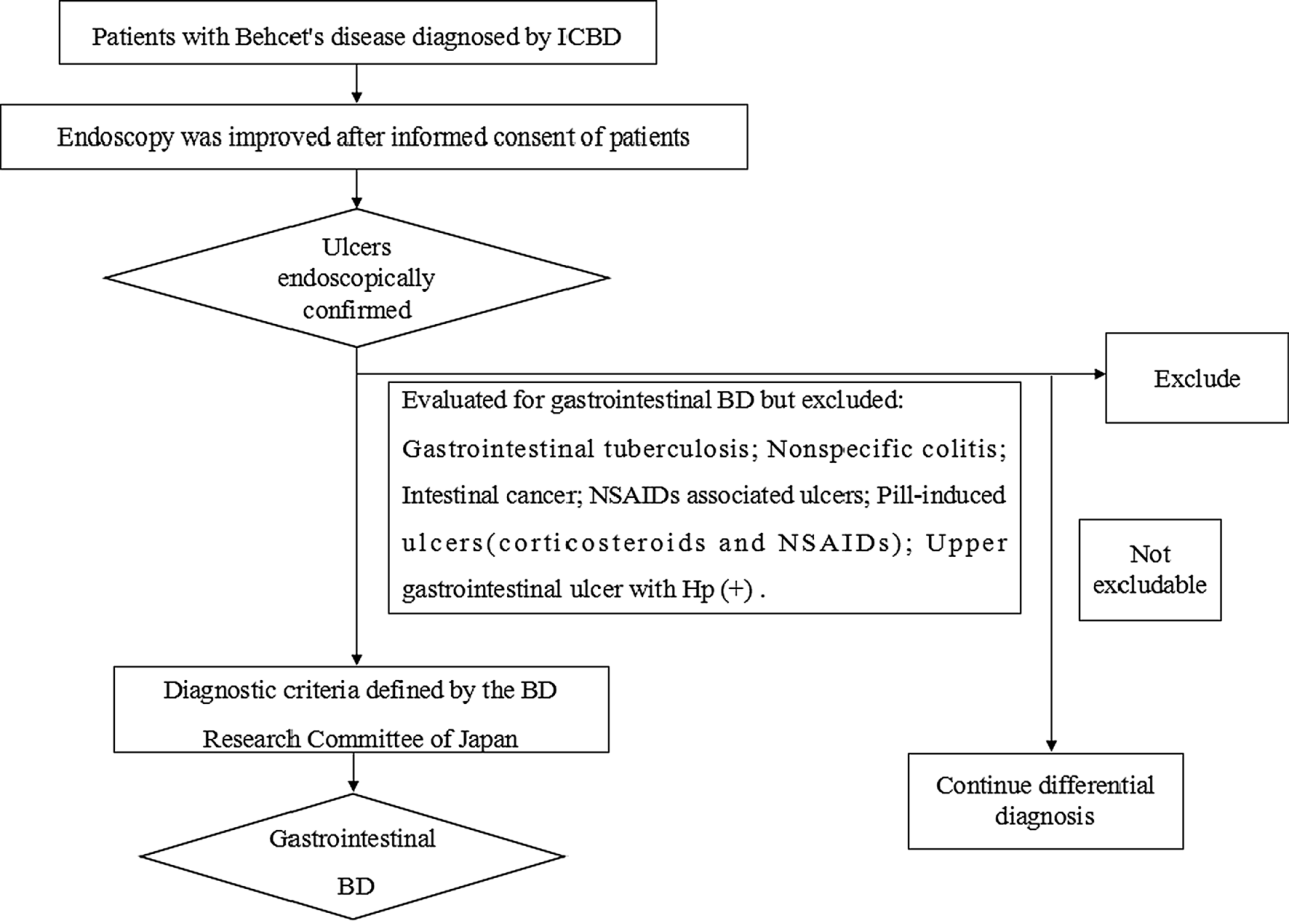
Algorithm for a definite diagnosis of gastrointestinal BD

Data from the medical records of 1232 BD patients who underwent endoscopy between October 2013 and April 2020 were collected from Huadong Hospital affiliated to Fudan University. Of 1232 subjects, 273 were finally enrolled into the gastrointestinal BD group after meeting the following criteria: (1) Enrolled patients met the international Criteria for Behçet Disease (ICBD) published in 2013 [9]; (2) In all cases, inclusion in the study required that the existence of gastrointestinal ulcer be confirmed endoscopically or surgically; (3) Subjects with any other gastrointestinal diseases such as intestinal tuberculosis, nonspecific colitis, intestinal cancer or reflux esophagitis were excluded; (4) Patients were also excluded if they had any history of taking Non-Steroidal Anti-Inflammatory Drugs (including low doses of aspirin) within 30 days before endoscopy and there is peptic ulcer under endoscopy. (5) Those who met BD and had upper gastrointestinal ulcers were excluded if they were positive for Hp by rapid urease test under Endoscopy (Fig. 1).

We reviewed the medical records of 1232 subjects, recording demographics, clinical manifestations and laboratory findings including ESR, CRP and Hb. Clinical manifestations included gastrointestinal symptoms and various symptoms: fever, oral ulcers, genital ulcers, skin lesions, ophthalmitis, neurologic lesion, Myelodysplastic syndrome (MDS), venous thrombosis, valvulopathy and aneurysm. Disease locations and histopathology were also recorded in 273 BD patients with gastrointestinal ulcer. This study protocol was approved by the ethics committee of the hospital.

### Statistical analysis

Continuous variables were expressed as means with ranges. Discrete data were expressed as numbers or percentages or both. Demographics, clinical symptoms, and subjects with and without gastrointestinal ulcer in BD patients were compared. For comparative analyses, the Mann-Whitney U test and Student’s t test were used for continuous variables, while the chi-squared test was used for categorical variables, where appropriate. A logistic regression model for prediction of gastrointestinal ulcer was computed. All statistical analyses were performed using SPSS 17.0. P < 0.05 was considered statistically significant.

## Results

### The macroscopic and microscopic characteristics of gastrointestinal ulcers in BD patients (Table 1; Fig. 1)

**Fig. 2:**
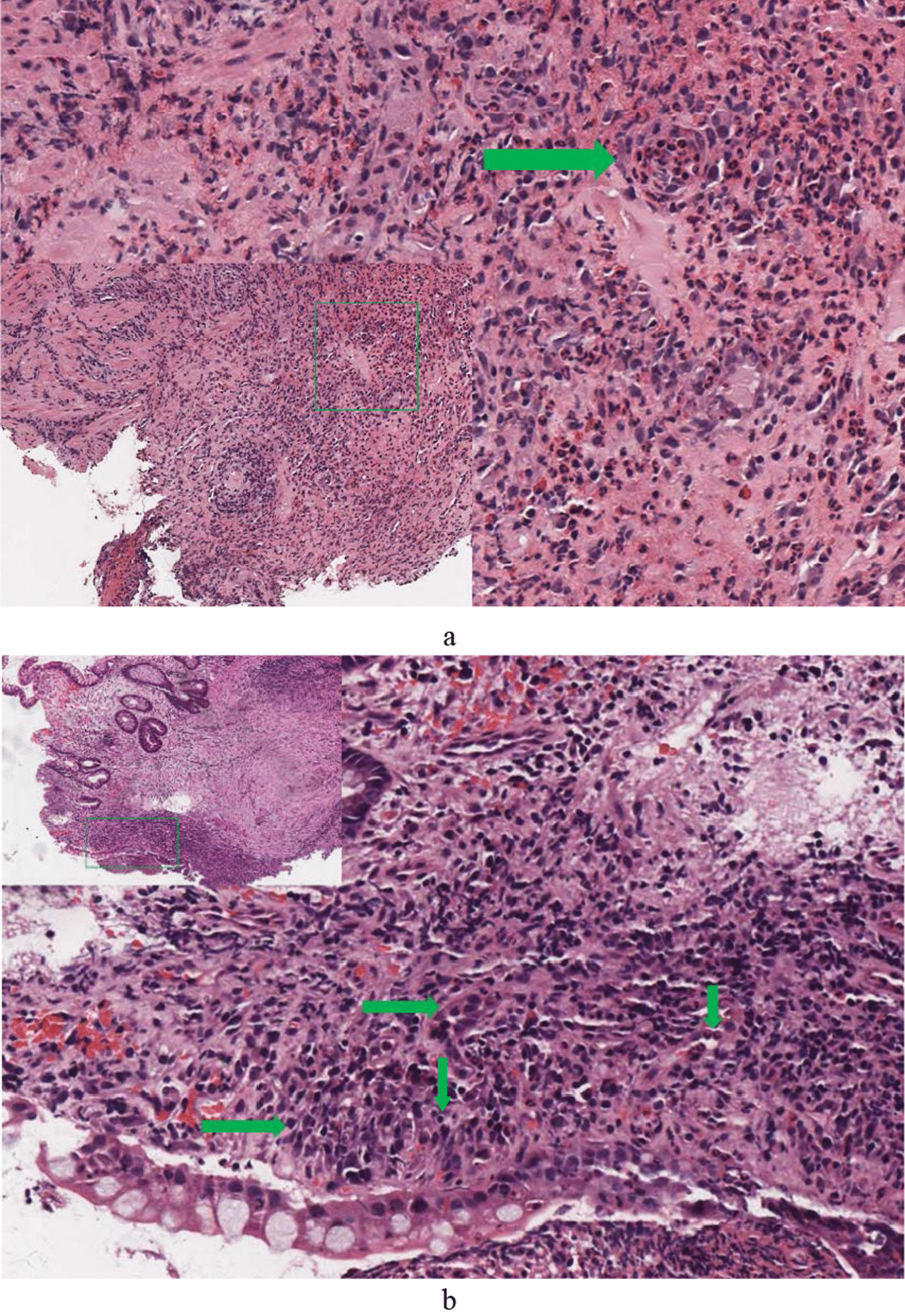
**a** A large number of neutrophils infiltrated into the small blood vessels in the lamina propria of esophageal mucosa, and the small blood vessels were inflamed. **b** Histopathologic findings in colonoscopic biopsy specimens demonstrating plasma cell infiltration, interstitial small vessel swelling, lymphocyte infiltration and neutrophil infiltration (arrow from left to right)

**Fig. 3:**
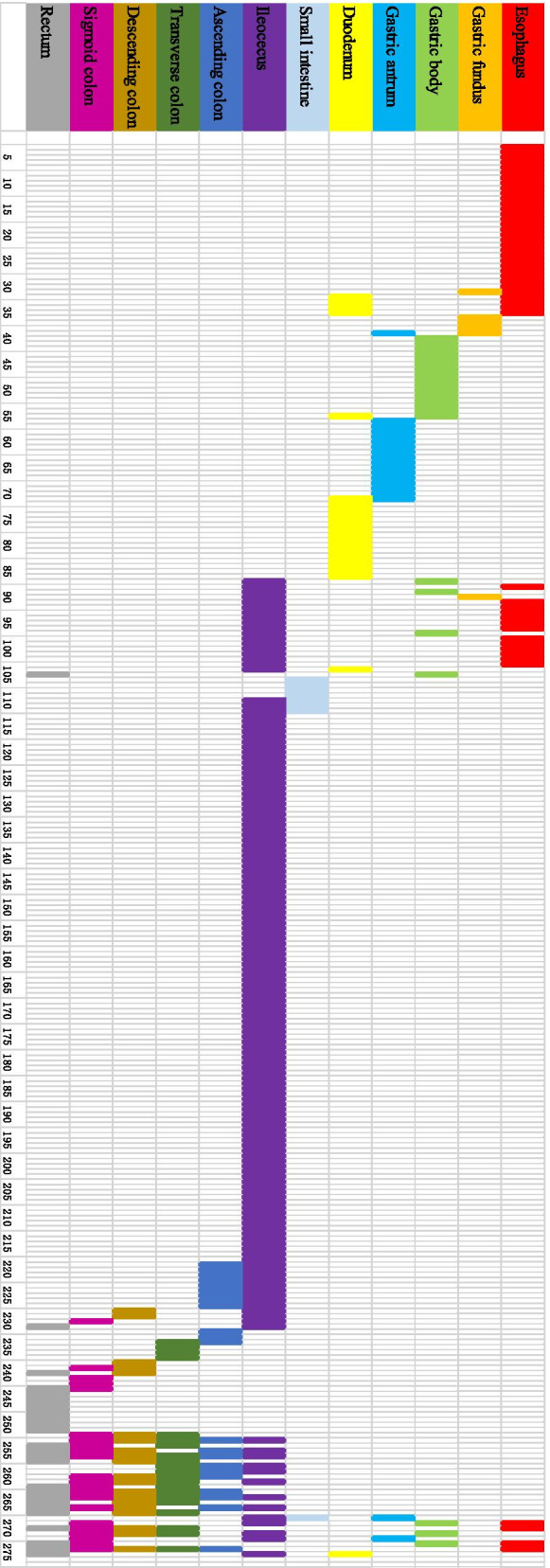
Distribution of gastrointestinal ulcer in 273 patients with BD. Endoscopy showed that gastrointestinal ulcer in patients with BD may occur in any part of the alimentary tract from the esophagus to the rectum. The main foci of the gastrointestinal ulcer in BD patients are typically the isolated terminal ileum and colon. The numbers of BD patients with gastrointestinal ulcers isolated to ileocecal region, ascending colon, transverse colon, descending colon, sigmoid colon and rectum were 106, 2, 3, 1, 2 and 8, respectively. However, isolated upper gastrointestinal tract involvement, including the esophagus, gastric body, gastric body, gastric antrum, and duodenum, lesions are also detected as well, with a relatively high frequency 30.76% (84/273). 24 patients with multiple segment involvement

Of 1232 BD patients, 22.16% (273/1232) had gastrointestinal ulcers. An almost equal distribution between females and males was found in the group of BD patients with gastrointestinal ulcer. The mean age was 39.33 ± 14.62 years. Gastrointestinal symptoms were noted in only 38.46% (105/273) in BD patients who had gastrointestinal ulcers confirmed by endoscopy or surgery. Of these, abdominal pain was the most common symptom 27.12% (74/273), followed by hematochezia 14.29% (39/273), diarrhea 12.09% (33/273), retrosternal pain 2.93% (8/273), dysphagia 2.56% (7/273), and nausea 0.37% (1/273). The diameter of ulcer ranged from needle-tip size to giant ulcer. Ulcers larger than or equal to 2 cm were recorded in 18 (22.22%) patients. Round/oval shape was the most common 99 (76.74%). Biopsy specimens were obtained from 183 patients. Inflammatory cell infiltration, including neutrophils, lymphocytes cells was the most common histopathologic feature 142 (77.60%), followed by granuloma 39 (21.31%). Vasculitis was found in only two patients (Table 1; Fig. 2a, b). The numbers of patients with BD had gastrointestinal ulcers isolated to the upper gastrointestinal tract, small bowel, ileocecal region, ascending colon, transverse colon, descending colon, sigmoid colon and rectum were 84, 4, 106, 2, 3, 1, 2 and 8, respectively. The numbers of intestinal segments involved in the remaining patients were more than or equal to 2. Of these, 24 patients had three or more intestinal segments involved. One patient suffered from total gastrointestinal tract involvement (Fig. 3).

**Table 1:** The characteristics of BD patients with gastrointestinal ulcer

Characteristics (n = 273)	
Age (years)	39.33 ± 14.62
Gender (male/female)	122/151
*Gastrointestinal symptom n (%)*	105 (38.46)
Abdominal pain n (%)	74 (27.12)
Diarrhea n (%)	33 (12.09)
Hematochezia n (%)	39 (14.29)
Nausea n (%)	1 (0.37)
Retrosternal pain n (%)	8 (2.93)
Swallow pain n (%)	7 (2.56)
No gastrointestinal symptom n (%)	168 (61.54)
*Morphology of ulcer*	
Approximate circle n (%)	99 (76.74)
Irregular shape n (%)	19 (14.73)
Annular ulcer n (%)	3 (2.33)
Longitudinal ulcer n (%)	8 (6.20)
Ulcer diameter > 2 cm n (%)	18 (22.22)
*Pathological findings*	
Vasculitis n (%)	2 (1.09)
Granuloma n (%)	39 (21.31)
Inflammatory infiltration n (%)	142 (77.60)

Values are presented as number (%) or mean ± standard deviation

#### Comparison of the demographic and clinical manifestations between BD patients with isolated upper gastrointestinal ulcers and isolated ileocecal involvement

We identified 84 BD patients with isolated upper gastrointestinal tract ulcer. Compared to controls with isolated ileocecal ulcers, these patients were younger at BD symptom onset (*P* = 0.008). An equal distribution between male and female was found, and no difference was noted between the two groups in terms of duration. The most frequent extraintestinal systemic manifestation of both groups was oral ulcer. Neurologic lesions were more frequent in BD patients with isolated ileocecal ulcers (*P* = 0.015), while skin lesions were more common in BD patients with isolated upper gastrointestinal tract ulcers (*P* = 0.035). The rates of complications including perforation (*P* = 0.004), obstruction (*P* = 0.015) and abdominal surgery at diagnosis were higher in BD patients with isolated ileocecal ulcers. There was no significant difference between the groups in terms of endoscopic pathological results, or levels of ESR, leukocytes, neutrophils, eosinophils, basophils, lymphocytes, monocytes, platelets, serum ferritin, and CRP (Table 2).

**Table 2:** Comparison between BD patients with isolated upper gastrointestinal ulcer and isolated ileocecus

Characteristics	BD patients with isolated upper gastrointestinal ulcer	BD patients with isolated ileocecus ulcer	*P*
Number of patients	n = 84	n = 106	
*Demographics*			
Gender (M/F)	55/29	56/50	0.054
Age at onset (years, mean±SD)	32.99 ± 14.36	27.33 ± 14.63	**0.008**
Duration (months, mean±SD)	108.70 ± 100.32	114.72 ± 122.27	0.716
*Clinical manifestations*			
Extraintestinal manifestations			
Oral ulcers n (%)	84 (100)	106 (100)	
Genital ulcers n (%)	57 (67.86)	71 (66.98)	0.512
Skin lesions n (%)	42 (50)	38 (35.85)	**0.035**
Ocular lesions n (%)	5 (5.95)	6 (5.66)	0.343
Neurologic lesions n (%)	0 (0)	7 (6.60)	**0.015**
Cardiac valvular lesions n (%)	1 (1.19)	0 (0)	0.442
Venous thrombosis n (%)	3 (3.57)	2 (1.89)	0.391
Aneurysms n (%)	3 (3.57)	1 (0.94)	0.229
Gastrointestinal complications			
Hemorrhage n (%)	7 (8.33)	15 (14.15)	0.155
Perforation n (%)	0 (0)	9 (8.49)	**0.004**
Obstruction n (%)	0 (0)	7 (6.60)	**0.015**
History of surgery n (%)	1 (1.19)	11 (10.38)	**0.008**
Assistant examination			
ESR (mm/h)	18 (7.25~28.75)	16 (7.50~40.00)	0.580
CRP (mg/L)	8.55 (3.19~10.00)	10 (3.68~19.65)	0.167
Leukocyte	6.06 ± 2.39	7.55 ± 3.18	0.084
Neutrophil	57.54 ± 15.38	63.11 ± 14.33	0.463
Eosinophil	2.36 ± 2.16	2.53 ± 1.48	0.220
Basophil	0.475 ± 0.37	0.47 ± 0.39	0.401
Lymphocyte	32.03 ± 12.62	26.43 ± 12.53	0.851
Monocyte	7.59 ± 3.03	8.35 ± 3.49	0.522
Platelet	231.91 ± 72.00	247.07 ± 89.09	0.277
Serum ferritin	185.31 ± 42.49	185.40 ± 44.17	0.985
BDCAF	3.28 ± 0.924	3.22 ± 1.146	0.100
Pathological findings			0.055
Vasculitis n (%)	0 (0)	2 (2.67)	
Granuloma n (%)	9 (15.25)	21 (28.00)	
Inflammatory infiltration n (%)	50 (84.75)	52 (69.33)	

*BD* Behçet’s disease, *SD* standard deviation

**P** values with statistical significance are shown in bold in the table

## Predictors associated with gastrointestinal ulcer in patients with Bechet’s disease

In a multivariate logistic regression model, gastrointestinal symptoms (OR 0.070, *P* < 0.001), fever (OR 0.115, *P* = 0.047), and high levels of CRP (OR 0.994, *P* = 0.027) and BDCAF level (OR 0.590, *P* = 0.010) were independent predictors for the presence of gastrointestinal ulcers. By contrast, Uveitis correlates negatively with gastrointestinal involvement in BD patients (OR 3.738, *P* = 0.011) (Table 3).

**Table 3:** Factors for prediction of gastrointestinal ulcer in BD patients

Variable	BD patients without gastrointestinal ulcer	BD patients with gatrointestinal ulcer	Univariate analysis, *P* value	Multivariate analysis, *P* value
Sex(F/M)	477:482	122:151	0.080	
Age at diagnosis	38.91 ± 13.53	39.33 ± 14.62	0.660	
Smoking history	87:872	28:245	0.312	
Archenteric symptoms	913:46	168:105	**< 0.001**	**< 0.001** **OR 0.070**
Oral ulcer n (%)	949 (98.96)	269 (98.53)	0.377	
Genital ulcer (%)	659 (68.72)	179 (65.56)	0.181	
Skin lesion (%)	471 (49.11)	117 (42.85)	**0.039**	n.s
Ocular (%)	164 (17.10)	16 (5.86)	**< 0.001**	**0.011** **OR 3.738**
Hematological (MDS/+8) (%)	9 (0.94)	12 (4.40)	**< 0.001**	n.s
Cardiac valvular lesions (%)	25 (2.61)	2 (0.73)	**0.041**	n.s
Venous thrombosis (%)	24 (2.50)	9 (3.30)	0.297	
Aneurysms (%)	32 (3.34)	7 (2.56)	0.338	
Fever (%)	9 (0.94)	32 (11.72)	**< 0.001**	**0.047** **OR 0.115**
ESR (mm/h)	12 (6 ~ 25)	19 (8 ~ 40)	**0.001**	n.s
CRP (mg/L)	6.5 (1.86 ~ 10)	9.7 (3.75 ~ 17.8)	**< 0.001**	**0.027** **OR 0.994**
Hemoglobin (g/L)	132.66 ± 17.44	127.69 ± 20.72	**< 0.001**	n.s
Leukocyte	9.47 ± 3.99	7.15 ± 3.20	0.280	
Neutrophil	59.05 ± 12.15	61.31 ± 14.91	**0.006**	n.s
Eosinophil	2.02 ± 1.72	1.73 ± 1.72	0.708	
Basophil	0.51 ± 0.46	0.61 ± 0.47	0.688	
Lymphocyte	30.24 ± 11.15	30.18 ± 12.85	**0.715**	
Monocyte	7.93 ± 2.88	8.20 ± 3.43	0.056	
Serum ferritin	144.67 ± 135.41	306.94 ± 198.77	**0.002**	n.s
Platelet	240.81 ± 82.84	240.81 ± 158.00	0.995	
BDCAF	2.86 ± 0.765	3.20 ± 1.07	**< 0.001**	**0.010** **OR 0.590**

*MDS* myelodysplastic syndrome

**P** values with statistical significance are shown in bold in the table

## Discussion

Behçet’s disease (BD) is a chronic multisystem complex disorder. It has been classified in multiple phenotypes according to organ involvement (mucocutaneous, ophthalmic, cardiac, vascular, neurological, hematological, and gastrointestinal) through cluster and association studies [10, 11]. Despite the fact that gastrointestinal ulcer is not part of the classification criteria for this disease, its incidence is not low and can easily lead to severe morbidity and mortality. BD is usually designated “entero-BD” if an ulcer in the gastrointestinal tract is identified, and is always classified as an independent phenotype [12]. To date, much research has been conducted to determine the involvement of the intestinal BD. Nevertheless, the full length of digestive tract in BD has not yet been studied extensively. In the present study, we investigated the clinical features and risk factors of BD with gastrointestinal ulcers.

We identified 273 (22.16%) patients with gastrointestinal ulcers, a higher proportion than reported previously [13]. We speculate that the differences are due to the fact that previous studies mostly involved endoscopy in symptomatic BD patients [14], in addition to being based on dissimilar subject baseline characteristics such as race, geography, and differences in diagnostic criteria [6]. Consistent with other studies [4, 11], abdominal pain, hematochezia, diarrhea, retrosternal pain, and nausea can occur in BD patients with gastrointestinal ulcer. However, the presence of gastrointestinal symptoms does not always accurately indicate the presence of gastrointestinal ulcer in BD patients. Similar phenomena do not occur in the Chinese population merely [15, 16]. Yi et al. reported that only half of the BD patients with upper gastrointestinal symptoms did actually have esophageal involvement [6]. Therefore, the ability of gastrointestinal symptoms to predict the presence of gastrointestinal ulcers is limited. We further analyzed the clinical risk factors of BD patients with gastrointestinal ulcer and found that the higher CRP and fever suggested the presence of gastrointestinal lesions. This is in accordance with results of a previous study [12]. Compared with patients without GI involvement, fewer ocular lesions, lower levels of albumin, erythrocyte counts and hemoglobin, and higher levels of CRP and ESR were found in the intestinal BD group [12]. These results may aid the establishment of guidelines for gastrointestinal examination among BD patients. In addition, a study of 43 patients with BD from Japan, Haruko et al. reported that BD with gastrointestinal ulceration had a lower frequency of eye involvement and higher incidence of arthritis and vascular involvement than did BD without gastrointestinal lesions [17]. We also found that BD patients with uveitis correlates negatively with gastrointestinal involvement. Uveitis may be a protective factor for gastrointestinal ulcer in patients with BD. These findings may not reveal any causal relationships. The association of clinical features revealed in this study does not imply a causal relationship. However, it may hide the conclusion that uveitis and gastrointestinal involvement may have different pathogeneses. This requires us to further explore the various etiopathogenesis mechanism of BD between different clinical phenotypes.

Numerous clinical studies have described the macroscopic manifestations of gastrointestinal ulcers in BD patients. A few (number of ulcers < 5), round shape, and discrete border are general characteristics of gastrointestinal ulcer with BD [18, 19], which is consistent with our findings. In addition to, we found that focal distribution is another macroscopic feature of gastrointestinal ulcers in BD [18, 20]. Despite the fact that ulcers can be seen at any portion of the gastrointestinal tract, diffuse and continuous colonic involvement is rare [21]. Upper gastrointestinal tract ulcers in BD have been documented mostly in anecdotal studies report [3–5]. In this study we found that upper gastrointestinal tract involvement may occur either isolated 6.82% (84/1232) or together with other digestive tube locations 1.62% (20/1232). Compared with the typical isolated ileocecal ulcerations, there were no significant differences in terms of demography, parenteral manifestations, pathological biopsy, or levels of erythrocyte sedimentation rate and C-reactive protein. While, the rates of perforation, obstruction and history of surgery were less common in isolated upper gastrointestinal tract than in the isolated ileocecal group, as reported previously [7]. The exact proportion of small-bowel involvement in BD has not been well determined. We found small intestinal ulcer can exist alone or with ileocecus, which is consist with the past study [22]. Therefore, evaluation of the small bowel by capsule endoscopy is also necessary in all patients BD.

BD is considered as a neutrophilic vasculitis and the role of neutrophils in BD pathogenesis has been repeatedly suggested in recent years. Elevated IL-17 causes significant neutrophil and lymphocyte influx in neutrophil-mediated inflammatory responses [23]. The retained fecal products result in the characteristic chronic granulomatous inflammation and adaptive immune responses. Impaired digestion of bacteria and fungi by neutrophils can result in similar pathological and clinical pictures [24]. In our study, histopathologic examination of the biopsy specimens revealed signs of vasculitis in only two patients. The distribution of this pathological result may be related to superficial and narrow colonoscopic biopsies. The characteristic histopathologic lesions of intestinal BD usually require analysis of deep and wide tissue for diagnosis [25]. These pathological findings indicate local inflammatory activity around the gastrointestinal ulcer.

We analyzed a large number of patients in a single-center BD cohort. Stringent inclusion and exclusion criteria, standardized enrolment and completion by both patients and physicians minimized the limitations of an observational data analysis. Nevertheless, there remain some limitations. First, it was a a cross-sectional observational, hospital-based study with potential bias in its design. Second, this paper mainly analyzed macroscopic characteristics of the special phenotype of BD. Further research is necessary to determine the true pathogeneses of these phenomena.

## Conclusions

Upper gastrointestinal tract ulcers in BD may present in the form of isolated segment involvement, with lower incidence of perforation, obstruction stenosis, and history of surgery than isolated ileocecal ulcer. Gastrointestinal symptoms are not necessary conditions for the presence of gastrointestinal ulcer in BD patients. Routine endoscopy is advisable irrespective clinical manifestations. If endoscopy is not available, BD patients who develop fever, digestive tract symptoms, and elevated CRP should be strongly recommended to avoid serious complications caused by delayed diagnosis and treatment.

## Data Availability

Please contact author for data requests.
